# Alterations in Cytosolic and Mitochondrial [U-^13^C]Glucose Metabolism in a Chronic Epilepsy Mouse Model

**DOI:** 10.1523/ENEURO.0341-16.2017

**Published:** 2017-03-09

**Authors:** Tanya S. McDonald, Catalina Carrasco-Pozo, Mark P. Hodson, Karin Borges

**Affiliations:** 1 Department of Pharmacology, School of Biomedical Sciences, University of Queensland, St. Lucia, QLD 4072, Australia; 2 Department of Nutrition, Faculty of Medicine, University of Chile, Santiago 8380453, Chile; 3 Metabolomics Australia, Australian Institute for Bioengineering and Nanotechnology, the University of Queensland, St. Lucia, QLD 4072, Australia; 4 School of Pharmacy, the University of Queensland, QLD 4072, St. Lucia, QLD 4072, Australia

**Keywords:** glucose, glycolysis, metabolism, mitochondria, seizure, TCA cycle

## Abstract

Temporal lobe epilepsy is a common form of adult epilepsy and shows high resistance to treatment. Increasing evidence has suggested that metabolic dysfunction contributes to the development of seizures, with previous studies indicating impairments in brain glucose metabolism. Here we aim to elucidate which pathways involved in glucose metabolism are impaired, by tracing the hippocampal metabolism of injected [U-^13^C]glucose (i.p.) during the chronic stage of the pilocarpine-status epilepticus mouse model of epilepsy. The enrichment of ^13^C in the intermediates of glycolysis and the TCA cycle were quantified in hippocampal extracts using liquid chromatography–tandem mass spectroscopy, along with the measurement of the activities of enzymes in each pathway. We show that there is reduced incorporation of ^13^C in the intermediates of glycolysis, with the percentage enrichment of all downstream intermediates being highly correlated with those of glucose 6-phosphate. Furthermore, the activities of all enzymes in this pathway including hexokinase and phosphofructokinase were unaltered, suggesting that glucose uptake is reduced in this model without further impairments in glycolysis itself. The key findings were 33% and 55% losses in the activities of pyruvate dehydrogenase and 2-oxoglutarate dehydrogenase, respectively, along with reduced ^13^C enrichment in TCA cycle intermediates. This lower ^13^C enrichment is best explained in part by the reduced enrichment in glycolytic intermediates, whereas the reduction of key TCA cycle enzyme activity indicates that TCA cycling is also impaired in the hippocampal formation. Together, these data suggest that multitarget approaches may be necessary to restore metabolism in the epileptic brain.

## Significance Statement

The specific metabolic impairments that occur in the epileptic brain and can play a role in the development of seizures are mostly unknown. Glucose uptake has been shown to be reduced in epileptic brain areas in patients and experimental models. By following ^13^C-glucose metabolism, we show that during the chronic epileptic stage in a murine model, there are further impairments to oxidative glucose metabolism along with reduced maximal activities of pyruvate dehydrogenase and 2-oxoglutarate dehydrogenase, key enzymes of the TCA cycle in the hippocampus. Together with diminished glucose uptake, this will decrease the ability to produce ATP in epileptogenic areas, which may contribute to seizure development. This research identifies targets for new therapies to inhibit seizures in the epileptic brain.

## Introduction

Temporal lobe epilepsy (TLE) is one of the most common forms of epilepsy in adults, with approximately one-third of patients being multidrug resistant. Many of the pathophysiological characteristics and chronic spontaneous seizures of TLE are reflected in rodents after pilocarpine-induced status epilepticus (SE; Borges et al., 2003). Epileptic disorders are often associated with genetic mutations (Mulley et al., 2005; Escayg and Goldin, 2010), inflammation (Vezzani et al., 2011), and an imbalance between excitatory and inhibitory neurotransmission (Avoli et al., 2016). In addition, there is growing evidence that dysfunction in metabolic pathways within brain tissue such as glycolysis, the TCA cycle, and electron transport chain contribute to the initiation and progression of seizures (Alvestad et al., 2011; Tan et al., 2015).

In patients with TLE, numerous positron emission tomography (PET) studies using ^18^F-labeled fluorodeoxyglucose (^18^FDG) have shown that during a seizure event, glucose uptake is increased, whereas less glucose is taken up interictally in the epileptogenic zone (Kuhl et al., 1980; Chugani and Chugani, 1999; Vielhaber et al., 2003). In the chronic rat lithium-pilocarpine model of epilepsy, local cerebral glucose utilization rates (LCMR_glcs_) were reduced in several brain regions in between seizures, including the hippocampal CA1 and CA3 areas, as determined by the use of [^14^C]2-deoxyglucose (^14^C-2DG; Dubé et al., 2001). The limitations of these studies are that after metabolism via hexokinase, the 6-phosphates of ^18^FDG and ^14^C-2DG are not substrates for subsequent glycolytic reactions. Thus, these studies cannot provide any indication relating to further changes in glucose metabolism.

The metabolism of glucose has previously been studied in both the pilocarpine- and lithium pilocarpine-induced SE rodent models. Elevated hippocampal glucose concentrations were observed in the chronic stage of the lithium pilocarpine rat model; however, no change was found in the concentrations of [1-^13^C]glucose (Melø et al., 2005). Despite this lack of change in [1-^13^C]glucose amounts, the concentrations of glutamate and GABA resulting from [1-^13^C]glucose metabolism were lower in the SE mice during the chronic phase. Similarly, in the mouse pilocarpine model, we found a lower-percentage enrichment of ^13^C derived from [1,2-^13^C]glucose metabolism in citrate, malate, and the amino acids GABA and aspartate, without a change in glucose concentrations or percentage enrichment of [1,2-^13^C]glucose (Smeland et al., 2013). Together, these results suggest that glucose metabolism is perturbed in chronic epileptic rodent models, which may be a result of recurrent seizures but also may contribute to seizure development.

Although previous studies have indicated a disturbance in glucose metabolism in the chronic epileptic brain, it is unclear where the perturbation in glucose metabolism occurs. Here, we performed a comprehensive study of glucose metabolism, using the mouse pilocarpine SE model to determine the changes that occur in hippocampal glucose metabolism during the chronic epileptic stage, with the use of [U-^13^C]glucose.

## Materials and Methods

### Animals

Male CD1 mice (Australian Research Council) were individually caged under a 12-h light-dark cycle with standard diet as used in previous studies (SF11-027, Specialty Feeds Hadera et al., 2013; McDonald et al., 2013) and water ad libitum. The animals were adapted to conditions for at least 1 week and were 7–8 weeks old when used in experiments. All efforts were made to minimize the suffering and number of animals used. All experiments were approved by the University of Queensland’s Animal Ethics Committee and followed the guidelines of the Queensland Animal Care and Protection Act 2001. This work was performed according to the ARRIVE guidelines (https://www.nc3rs.org.uk/arrive-guidelines).

### Pilocarpine status epilepticus model

As described previously (Smeland et al., 2013), mice were injected with methylscopolamine (2 mg/kg intraperitoneally in 0.9% NaCl; Sigma-Aldrich) 15 min before pilocarpine (345 mg/kg subcutaneously in 0.9% saline; Sigma-Aldrich). After a 90-min observation period, mice were injected with pentobarbital (22.5 mg/kg intraperitoneally in 0.9% NaCl; Provet) to stop SE. Mice were defined as developing SE if they were observed to have continuous seizure activity mainly consisting of whole-body clonic seizures. Those that did not display this behavior were classified as No SE.

### [U-^13^C]glucose injections and tissue extraction

Three weeks after SE, 10 SE mice and 11 No SE mice were injected with [U-^13^C]glucose (0.3 mol/L i.p., 558 mg/kg; 99% ^13^C; Cambridge Isotope Laboratories). To denature brain enzymes and other proteins immediately, mice were killed by focal microwave fixation to the head at 5 kW for 0.79–0.83 s (Model MMW-05, Muromachi) 15 min after [U-^13^C]glucose injections. Mice were then decapitated, and hippocampal formations were dissected out and stored at –80°C until extracted. Samples were sonicated in 1 ml of methanol using a Vibra Cell sonicator (Model VCX 750, Sonics and Materials) with 4 μL of a 1 mm azidothymidine (AZT) solution added as an internal standard. Polar metabolites were extracted from samples using a modified Bligh–Dyer water/methanol/chloroform extraction procedure at a 2/2/3 ratio as previously described (Le Belle et al., 2002). Samples were lyophilized, reconstituted, and stored at –80°C until analyzed.

### Liquid chromatography–tandem mass spectrometry

Intermediates of [U-^13^C]glucose were analyzed according to the method described in Medina-Torres et al. (2015) with modifications and additions to scheduled multiple reaction monitoring (sMRM) transitions to account for variable carbon labeling patterns. These sMRM transitions for all the unlabeled metabolites and their associated instrument parameters are detailed in Table 1.

**Table 1.** Analyte-dependent parameters for the transitions used in scheduled multiple reaction monitoring data acquisition

Analyte	Q1 (Da) ^12^C analyte	Q3 (Da) ^12^C analyte	RT (min)	DP (volts)	CE (volts)	CXP (volts)
Glucose 6-phosphate	258.89	96.7	8.1	–20	–30	–15
Fructose 6-bisphosphate	259.02	96.8	9.6	–20	–30	–15
Fructose 1,6-bisphosphate	339.08	96.9	21.9	–20	–30	–15
Dihydroxyacetone phosphate	168.84	97	11.9	–50	–14	–5
2- and 3-Phosphoglycerate	184.91	97	21.5	–50	–20	–5
Phosphoenolpyruvate	166.83	79	22.3	–40	–18	–5
Pyruvate	87.02	43	11.9	–45	–12	–1
Citrate	190.96	110.9	22.6	–50	–18	–7
Aconitate	172.94	84.9	22.6	–30	–18	–5
2-Oxoglutarate	144.95	100.8	20.5	–40	–12	–5
Succinate	117	73	18.5	–45	–16	–3
Fumarate	115.01	70.9	21.1	–45	–12	–1
Malate	133	70.8	19.7	–40	–22	–3

### Analysis of incorporation of ^13^C in glycolytic and TCA cycle intermediates

**Fig. 1.**
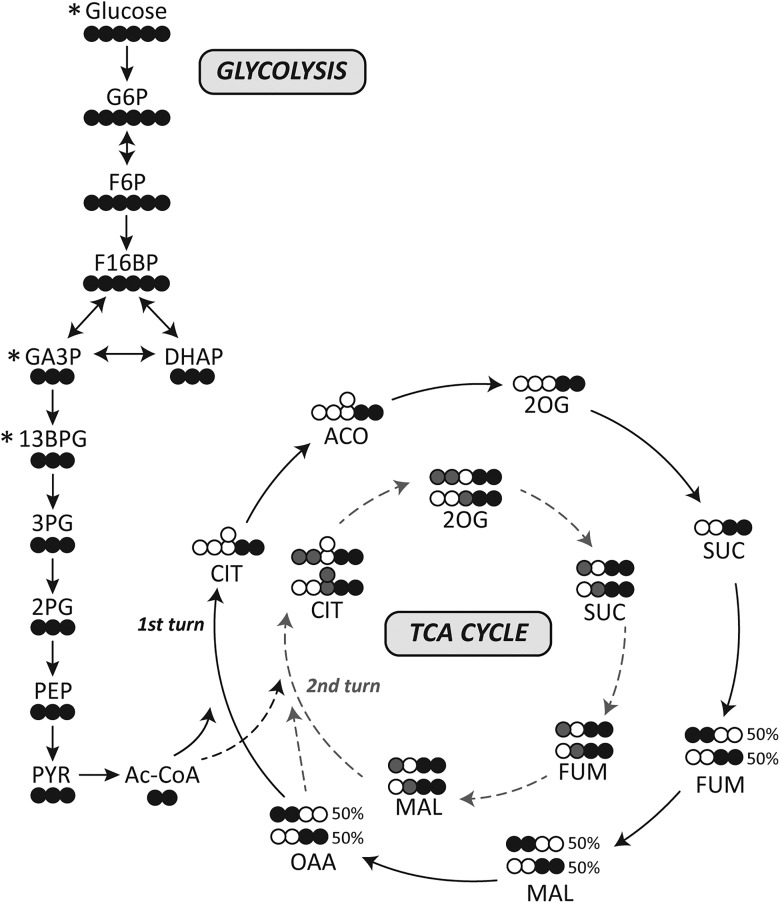
Schematic of [U-^13^C]glucose in the brain. Simplified schematic of ^13^C-labeling patterns after the metabolism of [U-^13^C]glucose via glycolysis and the TCA cycle. Empty circles, ^12^C; black filled circle, ^13^C; gray filled circles, ^13^C derived from ^13^C-labeled oxaloacetate that enters the second turn of the TCA cycle (gray dotted lines). *Metabolites that were not measured in this study. GA3P, glyceraldehyde 3-phosphate; 13BPG, 1,3-bisphosphoglycerate; 3PG, 3-phosphoglycerate; 2PG, 2-phosphoglycerate; Ac-CoA, acetyl CoA; OAA, oxaloacetate.

[U-^13^C]glucose can enter both neurons and astrocytes via glucose transporters GLUT3 and GLUT1, respectively. Once inside the cell, [U-^13^C]glucose is phosphorylated to [U-^13^C]glucose 6-phosphate, which can continue through the glycolytic pathway, producing glycolytic intermediates that are all uniformly labeled as shown in Fig. 1. These glycolytic intermediates can be measured using liquid chromatography–tandem mass spectrometry (LC-MS/MS) by first isolating the precursor ion (Q1 mass, Da) that is uniformly labeled with ^13^C. The masses isolated are glucose 6-phosphate (G6P), 265; fructose 6-phosphate (F6P), 265; fructose 1,6-phosphate (F16BP), 345; dihydroxyacetone phosphate (DHAP), 172; the combined metabolites of 2- and 3-phosphoglycerate (2 + 3PG), 188; phosphoenolpyruvate (PEP), 170; and pyruvate (PYR), 90. After collision-induced dissociation (Q2) the product ion detected (Q3 mass) for most glycolytic metabolites was dihydrogen phosphate ion (97 Da). For PEP, the product ion detected was a phosphate ion (79 Da), and PYR loses a carboxyl group, resulting in a detectable mass of 45 Da.

[U-^13^C]pyruvate resulting from glycolysis can produce [U-^13^C]lactate or alternatively enter the TCA cycle via pyruvate dehydrogenase (PDH, EC 1.2.4.1) to [1,2-^13^C]acetyl CoA. This entry of ^13^C-labeled acetyl-CoA results in two ^13^C carbons in all TCA cycle metabolites (Fig. 1). Thus, M + 2 isomers are isolated as the precursor ions (Q1, Da), for citrate (CIT),193; aconitate (ACO), 175; 2-oxoglutarate (2OG), 147; succinate (SUC), 119; fumarate (FUM), 117; and malate (MAL), 135. In the collision cell, all TCA cycle intermediates lose the carboxyl group. As the ^13^C is within one of the carboxyl groups of all metabolites after the collision, either one or two ^13^C-carbons remain on the product ion (Q3). Thus molecular weight (Da) of the product ions are 112 (lost a ^13^C in the collision, M + 1) and 113 (both ^13^C remain, M + 2) for CIT; 85 and 86 for ACO; 102 and 103 for 2OG; 72 and 73 for FUM; and 72 and 73 for MAL. The sum of both product ions’ percentage enrichment is representative of the first turn of the TCA cycle.

After the first turn, the resultant [1,2-^13^C]oxaloacetate or [3,4-^13^C]oxaloacetate can again condense with [1,2-^13^C]acetyl CoA (Fig. 1). This results in M + 4 CIT, which can be detected as above with the ions 195 (Q1), and then Q3 is either 114 (M + 3) or 115 (M + 4). Through the conversion of CIT to 2OG, a ^13^C may be lost and thus the precursor ion for 2OG will be M + 3 (148 Da, Q1 ion; 103 or 104 Da, Q3 ions). Alternatively, all four ^13^C carbons will be retained, resulting in an M + 4 precursor ion with both carboxyl groups containing a labeled carbon, one of which will be lost in the collision cell (149 Da, Q1; 104 Da, Q3). The remaining intermediates SUC, FUM, and MAL that can be measured will all contain three labeled carbons, with the possibility of retaining all or losing one ^13^C after the collision. Therefore, the molecular weight of the precursor ions isolated are 120, SUC; 118, FUM; and 136, MAL; with then 75 and 76 Da ions detected in Q3, and 73 and 74 Da for both FUM and MAL.

### Enzyme activities

Mice were decapitated under light isoflurane anesthesia. Brains were removed and hippocampal formations dissected out and stored at –80°C until used. Mitochondria were isolated as previously described (Tan et al., 2016). Aliquots were stored at –80°C and used to determine mitochondrial enzyme activities.

The activities of all enzymes were measured with the Spectromax 190 Microplate reader (Molecular Devices) via continuous spectrophotometric assays. All enzyme activities were normalized to protein content, measured via a Pierce bicinchoninic acid (BCA) assay (Thermo Fisher Scientific).

Hexokinase (HK, EC 2.7.1.1), phosphoglucose isomerase (PGI, EC 5.3.1.9), and glucose 6-phosphate dehydrogenase (G6PDH, EC 1.1.1.49), phosphofructokinase (PFK, EC 2.7.1.11), pyruvate kinase (PK, EC 2.7.1.40), lactate dehydrogenase (LDH, EC 1.1.1.27), and citrate synthase were measured as previously described (Tan et al., 2016). PDH (EC 1.2.4.1) was measured using the 3-(4,5-dimethyl-2-thiazolyl)-2,5-diphenyl-2H tetrazolium bromide (MTT) and phenazine methosulfate method (Ke et al., 2014).

Several enzyme activities were measured through the oxidation of reduced β-nicotinamide adenine dinucleotide (β-NADH) including glutamate dehydrogenase (GLDH, EC 1.4.1.2), glutamic pyruvic transaminase (GPT, EC 2.6.1.2), and glutamic oxaloacetic transaminase (GOT, EC 2.6.1.1). The GDH assay was initiated with 10 mm 2-OG, added to a reaction mix containing 100 mm potassium phosphate (pH 7.4), 100 mm ammonium chloride, and 0.6 mm β-NADH. GPT was measured in 100 mm triethanolamine buffer (pH 7.4), 0.6 mm β-NADH, 50 mm 2-OG, and 10 U/mL LDH (L2500, Sigma-Aldrich). GOT activity was measured in 80 mm Tris HCl (pH 7.8), 0.6 mm β-NADH, 15 mm 2-OG, and 5 U/mL malic dehydrogenase (M1567, Sigma-Aldrich) and initiated with the addition of 10 mm aspartate.

The activity of 2-oxoglutarate dehydrogenase (2-OGDH) was measured via the reduction of β-nicotinamide adenine dinucleotide (β-NAD^+^) in 75 mm Tris HCl (pH 8), 1 mm ethylenediaminetetraacetic acid, 0.5 mm thiamine pyrophosphate, 1.5 mm coenzyme A, 4 mm β-NAD^+^, 1 mm DTT, and 2 mm calcium chloride, and initiated with 15 mm 2-OG. Pyruvate carboxylase (PCX) activity was measured through the production of TNB^2+^ at a wavelength of 412 nm. The reaction mix contained 50 mm Tris HCl (pH 8), 50 mm sodium bicarbonate, 5 mm MgCl_2_, 5 mm sodium pyruvate, 5 mm ATP, 0.5 mm 5,5′-dithiobis-(2-nitrobenzoic acid), and 5 U/mL citrate synthase (C3260, Sigma-Aldrich), and the reaction was initiated with 0.1 mm acetyl CoA.

### Mitochondrial coupling assay

Using the extracellular flux XFe96 Analyzer (Seahorse Bioscience), the degree of coupling between the electron transport chain, the oxidative phosphorylation machinery, and ATP production was evaluated as previously described (Carrasco-Pozo et al., 2015; Tan et al., 2016). The contribution of nonmitochondrial respiration to oxygen consumption rate (OCR) was subtracted from every mitochondrial function parameter. Respiration linked to ATP synthesis was calculated as state 3 ADP – state 4o. All mitochondrial function parameters were normalized to protein content measured using a Pierce BCA protein assay kit.

### Mitochondrial electron flow

The sequential electron flow through the complexes of the electron transport chain was studied using the extracellular flux XFe96 Analyzer as previously described (Carrasco-Pozo et al., 2015). This assay allows study of the contribution and function of complexes I and II in the electron transport chain in terms of OCR. From the results, the complex I–driven (state 3u – OCR after rotenone injection) and complex II–driven (OCR after SUC injection – OCR after malonate injection) respiration were calculated.

### Data analysis

All statistical analyses were performed using GraphPad Prism version 6.0 (GraphPad Software). Two-way ANOVA followed by uncorrected Fisher’s least significant differences posttests were used for the total metabolite concentrations and percentage enrichment comparisons. Correlation analysis was performed to assess the correlation of percentage ^13^C enrichment of G6P to downstream glycolytic intermediates and the percentage enrichment of PYR relative to TCA cycle intermediates enrichment. Enzyme activities and functional mitochondrial parameters were analyzed using unpaired, two-sided Student’s *t*-tests. *p* < 0.05 was regarded as significant. All data are represented as mean ± SEM.

## Results

To assess the effects of pilocarpine-induced SE on brain glucose metabolism in mice in the chronic stage of the model, the total concentrations of glycolytic and TCA cycle intermediates were measured using LC-MS/MS, along with the percentage incorporation of ^13^C from i.p. injected [U-^13^C]glucose. Furthermore, mitochondrial electron transport functions were analyzed, and the activities of enzymes involved in all pathways were measured using spectrophotometric assays.

Of the 25 mice that were injected with pilocarpine, 12 (48%) developed SE, classified as continuous whole-body clonic seizures. Eleven (44%) did not develop these seizures and thus were classified as No SE, and two (8%) mice died from a seizure during the 90-min observation period. From the 12 mice that developed SE, 2 were killed in the following 3 d per ethical guidelines, as they did not recover well from SE.

In this study, we injected mice 3 weeks after SE in the chronic stage of the model with [U-^13^C]glucose to obtain information about glucose metabolism in the glycolytic and TCA cycle pathways. At this time point, the body weights of SE mice used for the [U-^13^C]glucose analysis were similar to the No SE group (39.9 ± 1.3 versus 39.8 ± 0.8 g, *p* = 0.97). Therefore, any changes in the total concentrations or percentage of ^13^C enrichment in brain metabolites are not due to differing amounts of [U-^13^C]glucose injected. No behavioral seizures were observed before or during the [U-^13^C]glucose injection until death. The total concentrations of metabolites in the glycolytic pathway and TCA cycle were similar among mice that had developed SE compared with those that did not, as shown in Table 2.

**Table 2.** Total levels of metabolites

Metabolite (nmol/g tissue)	No SE (*n* = 6–9)	SE (*n* = 6–7)
Glucose 6-phosphate	20.1 ± 1.7	24.2 ± 3.4
Fructose 6-phosphate	33.0 ± 2.2	36.2 ± 5.9
Fructose 1,6-bisphosphate	16.5 ± 1.0	17.8 ± 1.4
Dihydroxyacetone phosphate	0.70 ± 0.08	0.67 ± 0.08
2- and 3-Phosphoglycerate	11.2 ± 1.0	10.9 ± 1.2
Phosphoenolpyruvate	8.93 ± 1.42	7.50 ± 1.33
Pyruvate	38.2 ± 2.7	34.2 ± 6.0
Citrate	109 ± 5	110 ± 17
Aconitate	1.84 ± 0.12	2.24 ± 0.28
2-Oxoglutarate	90.8 ± 6.5	83.9 ± 17.4
Succinate	10.1 ± 0.8	8.1 ± 1.6
Fumarate	12.5 ± 0.9	13.2 ± 2.7
Malate	45.9 ± 3.9	46.1 ± 6.8

### Percentage enrichment of ^13^C in hippocampal glycolytic intermediates

**Fig. 2.**
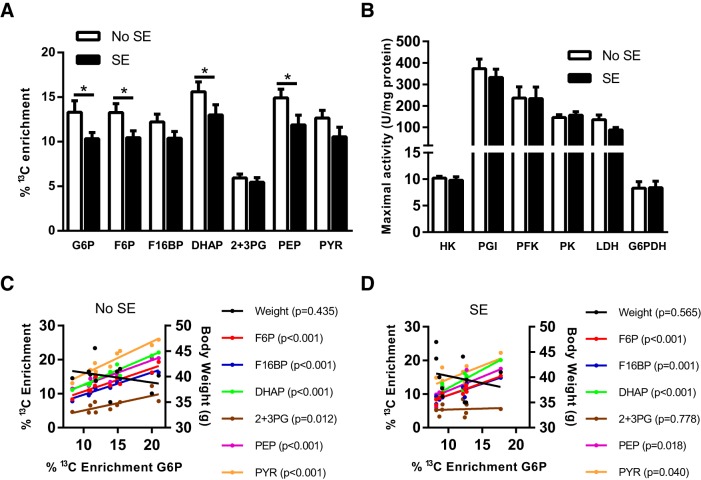
Metabolism of [U-^13^C]glucose via glycolysis in SE mice in the chronic stage of pilocarpine model. ***A***, Hippocampal ^13^C enrichment of glycolytic metabolites after i.p. injection of [U-^13^C]glucose was compared between SE and No SE mice. Reduced ^13^C enrichment in SE mice was found in G6P (22% reduction, *p* = 0.030), F6P (21%, *p* = 0.038), DHAP (17%, *p* = 0.05), and PEP (20%, *p* = 0.023). No significant differences were found in F16BP, 2 + 3PG, and PYR. Two-way ANOVA, SE status *p* < 0.001, *n* = 9–11 mice. ***B***, The activities of all cytosolic enzymes, namely HK, PGI, PFK, PK, LDH, and G6PDH, were unaltered between control No SE mice and mice after SE within the chronic stage of the model (*p* > 0.05 for all); *n* = 7–9. ***C***, Correlation analysis between percentage ^13^C enrichment of G6P and ^13^C enrichment in downstream metabolites in No SE mice. A significant correlation was observed with each metabolite, specifically: F6P, *r* = 0.89, *p* < 0.001; F16BP, *r* = 0.97, *p* < 0.001; DHAP *r* = 0.96, *p* < 0.001; 2 + 3PG, *r* = 0.76, *p* < 0.01; PEP, *r* = 0.96, *p* < 0.001; and PYR, *r* = 0.95, *p* < 0.001. No significant correlation was found between body weight (g) and percentage ^13^C enrichment of G6P, *r* = –0.28, *p* > 0.05. ***D***, Correlation analysis between percentage ^13^C enrichment of G6P and ^13^C enrichment in downstream metabolites in SE mice. Similar to the No SE group, a strong correlation was observed with each downstream metabolite apart from 2 + 3PG. F6P, *r* = 0.91, *p* < 0.001; F16BP, *r* = 086, *p* < 0.001; DHAP, *r* = 0.90, *p* < 0.001; 2 + 3PG, *r* = 0.10, *p* > 0.05; PEP, *r* = 0.72, *p* < 0.05; and PYR, *r* = 0.64, *p* < 0.05. No significant correlation was found between body weight (g) and percentage ^13^C enrichment of G6P, *r* = –0.21, *p* > 0.05.

As shown in Fig. 2*A*, the chronic stage after SE has an effect on the percentage enrichment of ^13^C in the chronic stage of the pilocarpine model (two-way ANOVA, *p* < 0.001). Specifically, reductions were found in the ^13^C enrichment of G6P (22%), F6P (21% reduction), DHAP (17%), and PEP (20%) in the SE mice compared with those that did not develop SE (*n* = 10–11, *p* < 0.05–0.01 for each metabolite in Fisher’s LSD posttest). No other significant differences were found in the percentage enrichment in other glycolytic intermediates, including fructose 1,6-bisphosphate (F16BP), PYR, and 2 + 3PG (*p* > 0.05, *n* = 10–11). The percentage ^13^C enrichment in all glycolytic intermediates are highly correlated with the percentage enrichment of the first metabolite of the pathway, G6P, in No SE mice (*r* = 0.76–0.97, *p* < 0.05–0.001, Fig. 2*C*). In contrast, no correlation was observed between the body weight of mice and the incorporation of ^13^C in G6P (*r* = –0.28, *p* > 0.05). Fig. 2*C* shows that this correlation was also observed in SE mice for all metabolites (*r* = 0.64–0.91, *p* < 0.05–0.001) apart from 2 + 3PG (*r* = 0.10, *p* = 0.78). Similarly, no correlation was observed between body weight and percentage ^13^C enrichment in G6P (*r* = –0.21, *p* > 0.05). This suggests that after the conversion of glucose to G6P, there is no alteration in the activity of the glycolytic pathway itself, but rather that glucose uptake is diminished in SE mice. No significant differences were observed between the body weight of either No SE or SE mice and the percentage ^13^C enrichment of G6P (Fig. 2*C*, No SE, *r* = –0.28, *p* > 0.05; Fig. 2*D*, SE, *r* = –0.21, *p* > 0.05).

The maximal activities of all cytosolic enzymes involved in the glycolytic pathway, namely PGI, PFK, and PK, were unaltered between No SE and SE mice in the chronic epileptic stage (Fig. 2*B*), which is consistent with the interpretation of results from the ^13^C analysis. No changes were found between the two groups regarding the activities of the other cytosolic enzymes LDH and G6PDH, responsible for the conversion of PYR to lactate and entry into the pentose phosphate pathway, respectively. It should be noted here that these enzymes, except G6PDH and PFK, are not rate limiting.

### Percentage enrichment of ^13^C in TCA cycle intermediates in the hippocampus

**Fig. 3.**
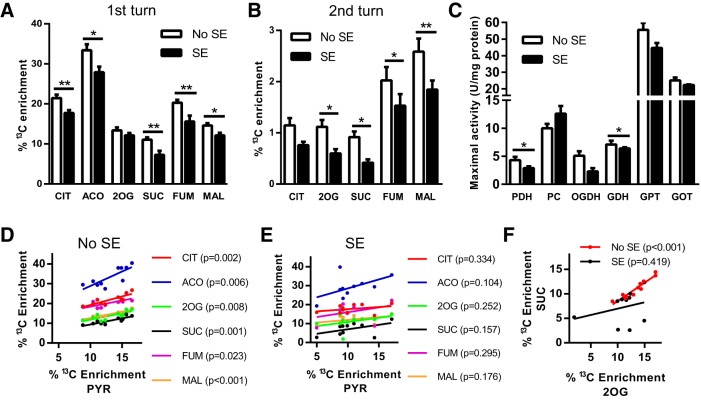
Metabolism of [U-^13^C]glucose via the TCA cycle is impaired in SE mice in the chronic stage of pilocarpine model. ***A***, Percentage ^13^C enrichment in the TCA cycle metabolites from the first turn of the TCA cycle were compared between SE and No SE mice. Reduced ^13^C enrichment was found in CIT (17% reduction, *p* < 0.006), ACO (17%, *p* = 0.0001), SUC (35%, *p* = 0.005), and FUM (23%, *p* = 0.001) in the hippocampal formation of mice in the chronic epileptic state. No changes were found in the ^13^C enrichment of 2OG (*p* > 0.05) or MAL (*p* > 0.05). Two-way ANOVA, SE status *p* < 0.001, *n* = 9–11 mice. ***B***, Percentage ^13^C enrichment of TCA cycle metabolites when labeled oxaloacetate condenses with [1,2-^13^C]acetyl CoA. A reduction in ^13^C enrichment was observed in the intermediates 2OG (47%, *p* = 0.03), SUC (55%, *p* = 0.037), FUM (25%, *p* = 0.044), and MAL (29%, *p* = 0.003). Two-way ANOVA, seizure status *p* < 0.001, *n* = 9–11 mice. ***C***, Maximal activities of mitochondrial enzymes were compared between SE and No SE mice. SE mice had lower activity of both PDH (33%, *p* = 0.045) and 2-OGDH (55%, *p* = 0.027), two key enzymes involved in the entry and rate of TCA cycling, compared with No SE controls. No changes were found in the enzymes PCX, GDH, GPT, and GOT (all *p* > 0.05); *n* = 7–9 mice for all enzymes. ***D***, Correlation analysis between percentage ^13^C enrichment in PYR to all first-turn TCA cycle intermediates in No SE mice. A significant correlation exists for all metabolites compared with PYR in this group. CIT, *r* = 0.86, *p* < 0.001; ACO, *r* = 0.80, *p* < 0.01; 2OG, *r* = 0.78, *p* < 0.01; SUC, *r* = 0.86, *p* < 0.01; FUM, *r* = 0.70, *p* < 0.05; and MAL, *r* = 0.91, *p* < 0.001. ***E***, Correlation analysis of percentage ^13^C enrichment in SE mice between PYR and first-turn TCA cycle intermediates. No significant correlation was found between PYR and the TCA cycle metabolites. CIT, *r* = 0.34, *p* > 0.05; ACO, *r* = 0.54, *p* > 0.05; 2OG, *r* = 0.40, *p* > 0.05; SUC, *r* = 0.48, *p* > 0.05; FUM, *r* = 0.37, *p* > 0.05; and MAL, *r* = 0.46, *p* > 0.05. ***F***, Correlation between percentage enrichment of ^13^C from the first turn of the TCA cycle between 2OG and SUC. A strong correlation was observed in ^13^C enrichment between the two metabolites in No SE mice (*r* = 0.95, *p* < 0.001), whereas no correlation was found in ^13^C enrichment of 2OG and SUC in SE mice (*r* = 0.42, *p* > 0.05).

The percentage enrichment of ^13^C in TCA cycle intermediates derived from [U-^13^C]glucose entering via pyruvate dehydrogenation were determined. We found a reduction in the percentage ^13^C enrichment in the TCA cycle intermediates CIT (17%), ACO (17%), SUC (34%), FUM (24%), and MAL (17%) in SE mice compared with No SE mice (all *p* < 0.05–0.01, Fig. 3*A*). 2OG was the only metabolite for which no significant change in ^13^C enrichment was observed between SE and No SE groups (*p* = 0.22).

The ^13^C-labeled oxaloacetate produced when [1,2-^13^C]acetyl-CoA enters the TCA cycle for the first time can be traced through the second cycle of the TCA cycle, if it condenses with ^13^C-labeled acetyl-CoA (Fig. 3*B*). Decreases in the percentage enrichment of ^13^C in the second turn of the TCA cycle were observed for 2OG (47%), SUC (54%), FUM (25%), and MAL (29%) in chronic SE mice (all *p* < 0.05–0.01). No change in percentage ^13^C enrichment was found in CIT (*p* > 0.05).

Correlations were observed between the ^13^C enrichments in PYR and those in first-turn TCA cycle metabolites resulting from PYR metabolism via PDH in No SE mice (*r* = 0.70–0.31, *p* < 0.01–0.001; Fig. 3*D*). This correlation was lost in SE mice (*r* = 0.34–0.54, *p* > 0.1–0.3), suggesting that there is another factor that determines entry of PYR into the TCA cycle in the chronic epileptic stage (Fig. 3*E*).

The maximal activity of the mitochondrial enzyme PDH, responsible for the entry of PYR into the TCA cycle, was reduced by 33% in chronic SE mice compared with No SE mice (Fig. 3*C*, *p* < 0.05). The maximal specific activity of OGDH, the rate-limiting enzyme of TCA cycling, was reduced by 55% in SE mice (*p* < 0.05). Similar activities were observed in the other mitochondrial enzymes PCX, GDH, GPT, and GOT (*p* > 0.05 for all enzymes), suggesting that the changes in PDH and OGDH activities were not due to loss of mitochondria. A strong correlation of the percentage enrichments within 2OG to those of SUC is observed in individual No SE mice (Fig. 3*F*, *r* = 0.95, *p* < 0.001), indicating that ^13^C enrichments of these two metabolites are highly dependent on each other. This correlation is lost in the SE mice, indicating that another factor such as the altered OGDH activity found plays a role (*r* = 0.29, *p* = 0.42).

### Mitochondrial coupling assays using extracellular flux

**Fig. 4.**
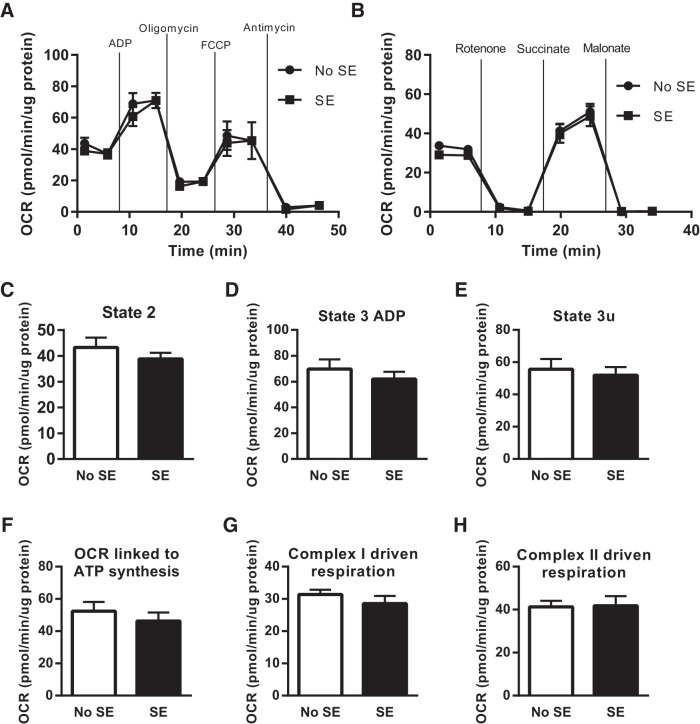
Mitochondrial functional parameters of isolated hippocampal mitochondria from SE and No SE mice measured with the extracellular flux analyzer. ***A***, Representation of the stages of the coupling assay to measure mitochondrial functions based on OCR. ***B***, An example of the stages of the electron flow assay to measure electron flow through the electron transport chain base on the OCR. No differences were found in any of the parameters measured using the coupling assay state 2 respiration (***C***), state 3 respiration after the addition of ADP (***D***), state 3 uncoupled respiration (***E***), and respiration associated with ATP synthesis (***F***). Similarly, no significant differences were observed in the parameters measured using the electron flow assay including complex I–driven respiration (***G***) and complex II–driven respiration (***H***) between No SE and SE mice (*n* = 6–8 mice).

Various functional parameters of the mitochondria isolated from the hippocampal formation were measured using the extracellular XF96 Analyzer. Similar results were observed in all functional parameters regarding the coupling assay (Fig. 4*A*) and the electron transport chain (Fig. 4*B*). This includes state 2, state 3 ADP, state 3u, and oxygen consumption linked to ATP synthesis (Fig. 4*C–F*). In addition, similar results were found in the complex I–driven and complex II–driven respirations of No SE and SE mice (Fig. 4*G*, *H*). Thus, there is no indication of general, mitochondrial dysfunction in the chronic epileptic brain in this mouse model.

## Discussion

Here we show direct evidence that glucose metabolism is lower in a chronic epilepsy mouse model because of the decrease in ^13^C incorporation into intermediates of both glycolysis (Fig. 2*A*) and the TCA cycle (Fig. 3*A*, *B*). Moreover, there was loss of activity in two rate-limiting enzymes of the TCA cycle, PDH and OGDH. No changes were found in the maximal activity of any enzymes involved in glycolysis. Last, similar rates of oxygen consumption were measured in hippocampal mitochondria from No SE and SE mice, indicating that the electron transport chain and ATP synthase are not affected in this model. Please note, we have previously shown using video–electroencephalography recordings that during the chronic phase of this model mice experience one to two spontaneous seizures a day (Benson et al., 2015). Mice were not experiencing behavioral seizures before or during death; thus these findings reflect changes in interictal glucose metabolism.

After the injection of [U-^13^C]glucose, the incorporation of ^13^C into several glycolytic intermediates was reduced in the hippocampal formation, including G6P, F6P, DHAP, and PEP. To our knowledge, no previous study has investigated the changes in glucose metabolism in chronic epilepsy via the quantification of glycolytic intermediates. Earlier studies have assessed lactate or alanine concentrations as indicators for changes in glycolysis with mixed results. In our earlier study in the same mouse model, there was no change in ^13^C enrichment in either lactate or alanine after injection of [1,2-^13^C]glucose (Smeland et al., 2013). Similarly, no alterations in the amounts of these intermediates were observed 24 h after kainate-induced SE in rats (Qu et al., 2003). However, reduced [3-^13^C]alanine from [1-^13^C]glucose metabolism was observed in the chronic lithium pilocarpine rat SE model, without a change in [3-^13^C]lactate concentrations (Melø et al., 2005). This was interpreted as defects in mitochondrial metabolism, as alanine can be metabolized in both mitochondria and the cytosol, whereas lactate is produced purely in the cytosol. Both lactate and alanine are products of PYR metabolism in the cytosol, whereas PYR also enters the mitochondria to produce products of the TCA cycle via PDH, PCX, or GPT. Therefore, a change in the concentrations of either alanine or lactate can be reflective of an alteration of glycolytic or TCA cycle activity that leads to an imbalance of the activities of these two pathways (Greene et al., 2003). Our current data of lowered enrichment of ^13^C in glycolytic intermediates in SE mice, together with our earlier result of unchanged [3-^13^C]lactate and [3-^13^C]alanine concentrations, indicate that less [U-^13^C]pyruvate must be metabolized to acetyl-CoA to maintain similar incorporation of the label into lactate and alanine compared with No SE mice. This is also corroborated by our finding of decreased PDH activity.

A limitation of this study was the inability to measure both the total concentration and the enrichment of ^13^C in glucose. Thus, we do not have any direct indications for potential alterations of glucose uptake by the epileptic brain, although previous studies showed reduced glucose uptake in adult rats in the chronic stage (see below). Because no changes were found in the activities of any regulatory enzymes in the glycolytic pathway, including HK, PFK, and PK, it is unlikely that glycolytic activity itself is impaired. Moreover, correlation analysis (Fig. 2*C*, *D*) of the ^13^C enrichment shows that in both No SE and SE mice there was a strong correlation between the enrichment of ^13^C in G6P and most downstream metabolites. Furthermore, a lack of correlation was evident between the body weight of mice, which determined the amount of [U-^13^C]glucose injected, and the ^13^C enrichment of G6P. Together this suggests that chronic epilepsy does not alter glycolysis, and thus the lower incorporation of ^13^C in SE mice is due to reduced uptake of glucose in the hippocampus, but not the activity of this pathway itself. This indicates that if glucose uptake was restored in this chronic epileptic state no impairment would be observed in the glycolytic pathway.

Several studies using ^18^FDG-PET have shown that interictal glucose uptake in patients is reduced (Henry et al., 1990, 1993; Arnold et al., 1996). Similarly, in the rodent lithium-pilocarpine model of epilepsy, glucose uptake is also reduced during the chronic phase (Dubé et al., 2001; Lee et al., 2012). Both these studies also provided evidence of neuronal loss in regions of reduced glucose uptake, which may at least in part be responsible for reduced glucose uptake. Hippocampal neuronal loss has previously been characterized in the mouse pilocarpine model (Borges et al., 2003) and may also contribute to the results of our study. However, several studies have failed to correlate neuronal loss with glucose metabolism (O’Brien et al., 1997; Dubé et al., 2001), suggesting that changes in interictal glucose metabolism are not wholly due to neuronal loss. Our study now provides the first evidence that although glucose uptake is reduced within the hippocampus of the chronic epileptic brain and less glucose overall seems to be metabolized, the glycolytic pathway itself is unimpaired.

The other key finding of this study is a reduction of ^13^C enrichment in TCA cycle intermediates after entry of [1,2-^13^C]acetyl CoA via PDH (Fig. 3*A*, *B*), as well as in the second turn of the TCA cycle. This can be partially explained by the reduced ^13^C enrichment in the glycolytic intermediates: there is less [1,2-^13^C]acetyl CoA available to form CIT. However, in No SE mice, ^13^C enrichment of PYR is highly correlated to ^13^C enrichment in TCA cycle metabolites from the first turn in the TCA cycle (Fig. 3*D*). This correlation is lost in SE mice, which suggests that in the chronically epileptic mice there are other factors that influence entry of PYR into the TCA cycle (Fig. 3*E*), such as the 33% reduction found in PDH activity (Fig. 3*C*). Consistent with this, patients with mutations in the PDH complex that lead to deficient activity are known to present with epileptic phenotypes (Kang et al., 2007; Barnerias et al., 2010).

In this study, we also observed a loss of 55% of the maximal activity of 2-OGDH, the rate-limiting enzyme of TCA cycling (Fig. 3*C*). This enzyme shares the E3 subunit, dihydrolipoamide dehydrogenase, with the PDH complex. This subunit is a flavin-containing protein, which reduces NAD^+^ to NADH through the transfer of reducing equivalents from the dihydrolyl moiety (Carothers et al., 1989). Heterozygous knockout of this protein in mice has shown to reduce activity of both PDH and OGDH complexes, and the mice are more prone to neurodegenerative disorders (Gibson et al., 2000). In autopsied patients with Alzheimer’s disease, the protein concentrations of all subunits of OGDH were reduced compared with control patients in the cortex, with the loss of the E3 subunit protein being restricted to the hippocampus (Mastrogiacomo et al., 1996). Reduced activity was found in several other neurologic disorders as previously summarized (Kish, 1997). In a separate study, the activities of both OGDH and PDH were reduced in autopsied Alzheimer’s disease patients and were correlated with the severity of the disease (Bubber et al., 2005). Although the mechanisms behind reduced PDH and OGDH activity are currently unknown, they may be potential new targets to increase energy metabolism in chronic epilepsy and neurodegenerative disorders.

The change in PDH and OGDH activities also supports the further reduction found in ^13^C enrichment in metabolites that entered the second turn of the TCA cycle produced when ^13^C oxaloacetate condenses with [1,2-^13^C]acetate (Fig. 3*B*). Together, these results demonstrate that TCA cycling is impaired in the hippocampus in the chronic stage of the pilocarpine model, which agrees with previous studies in both rat and mouse chronic SE models that show reduced incorporation of ^13^C from glucose metabolism into the amino acids glutamate, GABA, and aspartate (Qu et al., 2003; Melø et al., 2005; Smeland et al., 2013).

We found similar mitochondrial oxygen consumption rates related to proton leak, ATP synthesis, coupling efficiency, and respiratory control ratio (Fig. 4*C–F*), which indicates lack of mitochondrial dysfunction in the electron transport chain and its involvement in the final steps of oxidative phosphorylation in this chronic model of epilepsy. Mitochondrial dysfunction has been found acutely after both kainate- and pilocarpine- induced seizures (Chuang et al., 2004; Carrasco-Pozo et al., 2015). However, we have previously shown that this dysfunction is transient, as no changes were found in any functional parameters 48 h after SE (Carrasco-Pozo et al., 2015), which is further supported by our results during the chronic phase.

It is difficult to assess to what extent the impairments in TCA cycle activity found here are the result of chronic recurrent seizures. However, together with reduction in glucose uptake, reduced TCA cycling will result in less ATP production in the hippocampus. This is highly likely to contribute to the generation of seizures as well as seizure spread within the brain, as ATP is critical for most cellular functions and the maintenance of membrane potentials, and a loss of ATP can lead to hyperexcitability. This is evidenced by the proconvulsant effects of toxins blocking the respiratory chain and ATP production, such as 3-nitropropionic acid (Haberek et al., 2000), as well as by the many patients with epileptic seizures due to inherited TCA and respiratory chain enzyme deficiencies (Burgeois et al., 1992; Barnerias et al., 2010; Khurana et al., 2013).

## Conclusions

In the chronic epileptic stage, glycolytic enzymatic activities and the metabolism of G6P were unimpaired in the hippocampal formation. However, glucose uptake is likely to be reduced in mice in the chronic epileptic stage, which reduced the incorporation of ^13^C from i.p. injected [U-^13^C]glucose into glycolytic intermediates. Also, there was decreased PYR entry into the TCA cycle via PDH and reduced TCA cycling, including decreased activity of OGDH, in this chronic epilepsy model. Together, this will lead to reduced ATP production despite unaltered activity of the electron transport chain and ATP synthase in the hippocampus, which is likely to contribute to seizures. In summary, these data reveal several potential metabolic targets to inhibit seizure generation in an epileptic brain.

## Synthesis

Reviewing Editor: Maiken Nedergaard, University of Rochester Medical Center Decisions are customarily a result of the Reviewing Editor and the peer reviewers coming together and discussing their recommendations until a consensus is reached. When revisions are invited, a fact-based synthesis statement explaining their decision and outlining what is needed to prepare a revision will be listed below. The following reviewer(s) agreed to reveal their identity: Helle Waagepetersen, Mauro DiNuzzo

Our manuscript was well received by both reviewers. The reviewers agree that the study describes chonic glycolysis reduction in the epileptic brains. Accordingly, the title of the manuscript should be more specific and clear.

Reviewer #1:

The manuscript describes specific metabolic impairments occurring in the epileptic brain which may play a role in the development of seizures. The authors show that during the chronic epileptic stage in a murine model, there are in addition to glucose uptake impairments also reduced oxidative glucose metabolism along with reduced maximal activities of pyruvate dehydrogenase and 2-oxoglutarate dehydrogenase. In contrast the authors show no effect in function of the electron transport chain and oxidative phosphorylation. The manuscript is well written and present interesting and novel findings. However, I do specify points below that the authors should consider to revise.

Title:

"glucose handling" is an unspecific term to use, especially when it is followed by "reduce glucose metabolism". Thus, it reads: glucose handling reduces glucose metabolism which is difficult to follow.

Specific comments:

Line29 add "animal" in front of models.

Line 35: consider limiting the number of "new".

Line 61-63: This sentence need clarification. Not knowing the experimental details of the Melo study the sentence does not provide the reader with helpful information.

Line 61-69. Is a description of results from previous studies.

Line 69-71. Is what can be concluded based on the results described in the previous sentences.

Line 61-71. I suggest that the authors switch the focus towards what the data in these previous publications mean, and what can be concluded from them, instead of describing them. It is very difficult to people not used to these kind of studies to get information out of a reduction in concentrations of GABA or enrichment of citrate for instance.

Line 136. Rephrase, since pyruvate cannot produce lactate.

Line 195: From this paragraph it is not clear whether it is whole brain mitochondria or only from hippocampus, please specify more details. Mitochondrial coupling assay and Line 204: Mitochondrial electron flow. Consider to merge these two paragraphs.

Please note the substrates used. Since reductions in PDH and OGDH are found in enzyme activity assays the substrates used in the Seahorse experiments should be mentioned. It is interesting that no differences are found in basal respiration when these two enzymes are only 50 % of max activity. Consider in the results or discussion to reflect upon the lack of effects on oxygen consumption while these enzymes that produce the substrate of ETC are dramatically reduced. Could it be due to the use of very high substrate concentrations in the Seahorse exp?

Line 432-441, using the preparation of isolated mitochondria only from hippocampus it should be possible using appropriate substrate concentrations of pyruvate and glutamate to monitor a reduced basal respiration reflecting reduced activity of PDH and OGDH.

Line 298 + 310: The use of the word "similar" is confusing. I presume you mean similar between the groups but it could be different the activity of different enzymes. Consider rephrasing.

Line 308: is something missing in the title of this paragraph "using the extracellular flux ??"

Line 315: The Seahorse data cannot be used to conclude on general mitochondrial function. The assays can be used to conclude on the function of the electron transport chain and oxidative phosphorylation and the coupling of these two elements.

Line 322 PDH is not a TCA cycle enzyme.

Line 334: It is difficult to follow the descriptions of the findings of earlier studies. Maybe it helps to not mix the terms "enrichment", "amount" and "concentration". Note in line 339 that it is not mentioned if the "reduced [3-13C]-alanine" is enrichment, amount or concentration. Consider as suggested above for the introduction to have more focus on the conclusions of these studies.

Line 343-346:

Consider rephrasing:

"Both lactate and alanine are products of pyruvate metabolism in the cytosol, while pyruvate also enters the mitochondria to produce products of the TCA cycle via PDH, pyruvate carboxylase or glutamic pyruvic transaminase." It reads like alanine aminotransferase produce products of the TCA cycle. Moreover, it may be better using "....products of TCA cycle metabolism.."

Line 384-385:

AcetylCoA do not enter the TCA cycle via PDH. This sentence should be rephrased.

Line 420:

Consider to change the sentence to:

".....both rat and mice chronic SE models that show reduced oxidative metabolism of 13C glucose."

The use of the amino acids as reflections of TCA cycle activity confuses people that are not used to 13C NMR and MS data.

Line 425-426 For the data referred from Chuang et al and Carrasco-Pozo et al be specific instead of using the broad term "mitochondrial dysfunction" which include all functions of the mitochondria and therefore is potentially confusing in this context.

Line 440 a "cycle" is missing.

Line 450 Consider add to the sentence that it is due to limited availability of substrate in the form of NADH to complex I of the ETC.

Reviewer #2:

The authors investigated glucose metabolism during pilocarpine SE mouse model using LCMS as well as enzymatic assays. This is an interesting study because energy metabolism can contribute to seizures onset and possibly epileptogenesis. However, the results do not support any causality between changes in metabolite labeling/enzyme activity and SE, but only association. I have some observations that should be addressed to clarify some important points and put the findings into perspective.

The main finding is that decreases of 13C labeling in glycolytic and TCA cycle intermediates occur without any change in absolute concentration, which of course entails that the overall rate of glucose energy metabolism decreases. Interictal hypometabolism is a well-known phenomenon, and a reduced glucose uptake can well be the cause or the effect of a reduced metabolic rate. The authors suggest a causal relation, yet they did not attempt to speculate on the reasons for any putative decrease in glucose uptake, or, more generally, decrease in glucose availability. For instance, this might be due to impaired GLUT-mediated transport.

Anyway, since energy production is driven by energy consumption and since maximal enzyme activities and mitochondrial respiration are unaltered (but see below), any decrease in ATP levels as suggested by the authors is unlikely (e.g. ATP turnover not concentration is reduced; put differently, ATP consumption may decrease and so does ATP production).

The measured enzyme activities are, in fact, maximal activities, as the author state in "Results" (Page 11 line 266) but not in "Methods" (Page 7 line 171). It should be more clear that the measured parameters do not reflect the activity of the relevant brain enzymes in vivo, but only "capacity". The activity of virtually all enzymes is regulated by phosphorylation and/or allosteric mechanisms, and the actual conditions in vivo cannot be replicated in assays using saturating concentrations of substrates/cofactors and in the absence of other (potentially unknown or indirect) modulators. For example, the actual reaction rates in vivo can be maintained even when the maximal activity is reduced if the levels of modulators change accordingly. The authors should comment on this and make this limitation clearly understandable before concluding that the unaltered enzyme maximal activities are "consistent with interpretation of results from 13C analysis" (Page 11 lines 268-9). Specifically, the reported decreases in PDH and OGDH reaction rates (-33% and -55%, respectively) are difficult to reconcile with unchanged pyruvate and oxoglutarate absolute concentrations. The trends for anaplerotic changes in PC and GPT (though not significant, Figure 3C) might compensate for this, i.e. for de novo synthesis of glutamate.

Based on the above arguments, I also feel that the title of the paper is somewhat vague (e.g., "impaired glucose handling"). I suggest to focus on the glycolysis reduction (and not impairment), which adds important, incremental information about the characterization of interictal hypometabolism. Unfortunately this study does not provide data for identifyng the underlying mechanisms. Thus, reduced glucose uptake is not the only possible explanation. Indeed, a decrease in energy metabolism of glucose can be viewed under the perspective of increased, probably aberrant neuronal inhibition during the interictal phase.

Other minor comments

Page 1 line 14 (Abstract): Consider replacing "suggesting that glucose uptake is reduced" with "suggesting that brain glucose uptake and/or availability is reduced".

Page 3 line 74: Please replace "where" with "which pathways" or "where in the metabolic network" to make it clear to you refer to metabolic pathways.

Pages 5,6,7 (Section "Analysis of incorporation..."). Too long and too many information. Probably some details can be taken out.

Page 11 lines 253-260. Regarding the correlation with Glc-6-P, please present data for % enrichment in both NoSE and SE and then data for weight in both NoSE and SE (or alternatively before weight and then % enrichement).

In few occasions, the authors mention "rate-limiting" enzymes. Not all readers can immediately understand the significance of changes in rate-limiting versus non rate-limiting reactions.

Figure 1: An asterisk is probably missing in Ac-CoA and OAA.

The authors state that there were no seizures before and during experiment until sacrifice. Do the authors have a log of time from last seizure? That could be useful as a confound in correlation analysis. The authors may otherwise want to cite papers that did not find any correlation between hypometabolism and time from last seizure.

Page 16 lines 375-379: In addition to neuronal loss, the authors may want to mention astrocytic degeneration and proliferation. These are probably time-dependent processes and the authors should comment on the expected situation at 3 weeks after SE (which seems to be somewhat in the middle between latent and chronic phase).

Page 18 line 434: Is something missing in this sentence?

Page 20 lines 473-476: Reference Borges et al (a,b) is duplicate.
